# VIEWPOINT FROM THE INTRAMURAL RESEARCH PROGRAM

**DOI:** 10.1093/geroni/igz038.1643

**Published:** 2019-11-08

**Authors:** Luigi Ferrucci

**Affiliations:** Translational Gerontology Branch, Intramural Research Program, National Institute on Aging, National Institutes of Health, Baltimore, Maryland, United States

## Abstract

Dr. Ferrucci will review accomplishments of the Intramural Research Program and make projections for the future

